# Adapting Cognitive Behavioral Therapy for Adolescents in Iraq via Mobile Apps: Qualitative Study of Usability and Outcomes

**DOI:** 10.2196/67137

**Published:** 2025-04-11

**Authors:** Radhwan Hussein Ibrahim, Marghoob Hussein Yaas, Mariwan Qadir Hamarash, Salwa Hazim Al-Mukhtar, Mohammed Faris Abdulghani, Osama Al Mushhadany

**Affiliations:** 1 Department of Clinical Nursing Sciences, College of Nursing, Ninevah University, Kirkuk St, Mosul, 41001, Iraq, 964 7722112891; 2 College of Nursing, Al Kitab University, Kirkuk, Iraq; 3 Department of Clinical Nursing Sciences, College of Nursing, University of Mosul, Mosul, Iraq; 4 College of Medicine, Ninevah University, Mosul, Iraq

**Keywords:** cognitive behavioral therapy, CBT, psychotherapy, mHealth, app, adolescents, teenager, mental health, usability, engagement, anxiety, depression, user experience, UX, focus group, interview, digital health

## Abstract

**Background:**

Mental health challenges, including anxiety and depression, are increasingly common among adolescents. Mobile health (mHealth) apps offer a promising way to deliver accessible cognitive behavioral therapy (CBT) interventions. However, research on the usability and effectiveness of apps explicitly tailored for adolescents is limited.

**Objective:**

This study aimed to explore the usability, engagement, and perceived effectiveness of a mobile CBT app designed for adolescents, focusing on user experiences and mental health outcomes.

**Methods:**

A qualitative study was conducted with 40 adolescents aged 13‐19 years (mean age 15.8, SD 1.9 years; 18/40, 45% male; 22/40, 55% female) who engaged with a CBT app for 4 weeks. Mental health diagnoses included anxiety (20/40, 50%), depression (15/40, 38%), and both (5/40, 13%). Of these, 10 (25%) of the 40 participants had previous CBT experience. Feedback was gathered through focus groups and individual interviews, and thematic analysis identified key themes related to usability, engagement, and perceived effectiveness. Quantitative data on mood and anxiety scores were analyzed with paired *t* tests.

**Results:**

The mean usability score was 3.8 (SD 0.6), and the mean effectiveness score was 3.9 (SD 0.7). Older participants (aged 16‐19 years) reported significantly higher usability (mean 4.1, SD 0.4) and effectiveness scores (mean 4.3, SD 0.5) compared to younger participants (aged 13‐15 years) (*P*=.03). Females had higher usability (mean 4, SD 0.6) and effectiveness scores (mean 4.2, SD 0.7) than males (mean 3.6, SD 0.7, and mean 3.5, SD 0.8, respectively; *P*=.03). Participants with prior CBT experience had 2.8 times higher odds of reporting high usability scores (95% CI 1.6‐5; *P*=.002) and 3.1 times higher odds of reporting high effectiveness scores (95% CI 1.7‐5.6; *P*=.001). Usability challenges included complex navigation (20/40, 50%), interface design issues (12/40, 30%), and content overload (8/40, 20%). Factors positively influencing engagement were motivation driven by personal relevance (20/40, 50%) and gamification features (10/40, 25%), while lack of personalization (14/40, 35%) and external distractions (18/40, 45%) were significant barriers. Mood improvement (15/40, 38%) and learning new coping skills (12/40, 30%) were the most reported outcomes.

**Conclusions:**

The mobile CBT app shows potential for improving adolescent mental health, with initial improvements in mood and anxiety. Future app iterations should prioritize simplifying navigation, adding personalization features, and enhancing technical stability to support long-term engagement.

## Introduction

Mental health challenges among adolescents have become increasingly prevalent, with issues like anxiety, depression, and stress-related disorders affecting a significant portion of this population [1-4]. Adolescence is a critical developmental period marked by emotional, cognitive, and social changes, which can increase vulnerability to mental health disorders [5-7]. The World Health Organization estimates that up to 20% of adolescents experience mental health conditions, highlighting the urgent need for effective and accessible interventions [8-10].

In response to this growing demand, mobile health (mHealth) apps have emerged as a promising solution for delivering mental health care [11-14]. These apps offer a flexible, cost-effective, and private means for adolescents to access psychological interventions, which may otherwise be limited due to stigma, lack of resources, or geographical barriers [15-19]. Among the various therapeutic approaches, cognitive behavioral therapy (CBT) has proven to be particularly effective in addressing common adolescent mental health issues like anxiety and depression [20-24]. CBT focuses on helping individuals identify and modify negative thought patterns and behaviors, making it well suited for delivery via mobile platforms that offer interactive and self-guided modules [25-28].

Despite the promise of mobile CBT apps, there remains a significant research gap regarding their usability and effectiveness for adolescents [29,30]. Most existing studies focus on adult populations or general app evaluations without considering younger users’ unique needs and preferences [31,32]. Adolescents may have different expectations for user experience, engagement, and motivation when interacting with digital health tools [33,34]. Additionally, this age group’s developmental and emotional characteristics necessitate a design that fosters engagement and provides adequate support [35-39].

In Iraq, mental health services face significant barriers due to stigma, lack of resources, and geographical constraints, which limit adolescents’ access to traditional therapy. Postconflict instability, socioeconomic challenges, and strained health care systems have compounded mental health issues among Iraqi youth, creating a need for innovative solutions tailored to this population. Adolescents in Iraq, particularly those in low-resource settings, may be unable to access face-to-face therapy due to the limited availability of mental health professionals and the high costs associated with treatment.

While the potential of mobile CBT apps is evident, there is a lack of research on their usability and effectiveness among adolescents, particularly in regions like Iraq. This study aims to address this gap by exploring the usability, engagement, and outcomes of a mobile CBT app tailored specifically for adolescents in Iraq. The findings will provide valuable insights into how digital mental health tools can be optimized to better serve this vulnerable population in low-resource settings.

This study aimed to evaluate the usability, engagement, and perceived effectiveness of a mobile CBT app for adolescents, with a focus on user experiences and its impact on mental health outcomes.

## Methods

### Participants

The study recruited adolescents aged 13‐19 years who were experiencing mental health challenges, including anxiety and depression. A convenience sampling method was used to select participants from various sources, including local schools, mental health clinics, and online mental health communities. Participants were referred by counselors or health care providers and expressed interest in participating. Inclusion criteria required that participants had a self-reported diagnosis of anxiety or depression, regular access to a smartphone, and, for those aged <18 years, parental consent. A sample size of 40 adolescents was chosen for an in-depth qualitative analysis of diverse perspectives while maintaining manageability for detailed thematic analysis. The study was conducted in Mosul, Iraq, ensuring a regional context for the findings. Data confidentiality and participant anonymity were prioritized throughout the study. Each participant was assigned a unique identifier code used in place of personal information during data collection, transcription, and analysis. This ensured that no identifying details were linked to the responses. Informed consent was obtained from all participants, outlining their rights to privacy and the confidential handling of their data. Interview and focus group recordings were securely stored in encrypted files, accessible only to authorized research team members. All transcripts were deidentified prior to analysis to protect participant identities further, and findings were reported in aggregate to prevent any individual from being identified.

### Intervention

Participants were introduced to a CBT mobile app designed specifically for adolescents. The app was commercially available and selected based on its adherence to established CBT principles, as well as its focus on common adolescent mental health issues such as anxiety, depressive thoughts, and stress management. The app featured interactive modules, journaling functions, mood tracking, and self-assessment tools, all aimed at guiding users through CBT-based interventions. The selection of the app was influenced by its popularity, user ratings, and evidence-based framework, ensuring that it met the study’s requirements for delivering structured CBT exercises. Participants were asked to engage with the app for 4 weeks, with a recommendation to complete at least 3 CBT exercises per week, though they were free to use it at their discretion.

### Study Design

The study adopted a qualitative approach to gather rich, in-depth insights into the participants’ experiences with the mobile CBT app. Data were collected through focus groups and one-on-one semistructured interviews after the 4-week intervention period. This design allowed for exploring the app’s usability, engagement, and perceived outcomes from the adolescent perspective. The qualitative approach was well suited in understanding users’ subjective experiences and identifying themes related to app interaction and mental health improvement.

### Data Collection

Data collection focused on both app interaction and user feedback. The following methods were used.

#### App Usage Metrics

In-app data were collected to track the frequency of app usage, time spent on activities, completion of CBT modules, and interaction with various app features (eg, journaling and mood tracking).

#### User Feedback

Participants provided feedback through focus groups and interviews, where they discussed their experiences with the app. Topics of discussion included usability (ease of navigation and design preferences), engagement (motivation to use the app and consistency of use), and perceived outcomes (changes in mood, anxiety, or stress levels). Interviews were recorded and transcribed for analysis.

### Outcome Measures

The study evaluated the following outcomes.

#### Usability

This included ease of navigation, design intuitiveness, and app aesthetics. Participants shared feedback on the app’s user-friendliness and any barriers they encountered.

#### Effectiveness

Participants self-reported any changes in their mental health symptoms, particularly about anxiety and depression. Symptom reduction was assessed using qualitative descriptions of mood changes and mental health improvements throughout the study.

#### Overall Satisfaction

Participants reflected on their satisfaction with the app, including its features, content relevance, and overall impact on their mental health. Satisfaction was gauged through subjective feedback on whether they would recommend the app to peers or continue using it poststudy.

### Ethical Considerations

This study was conducted in accordance with the ethical guidelines set in the Declaration of Helsinki and was approved by the institutional review board of the University of Ninevah (approval reference number: NURIRB/041/2023). All the processes involving human subjects were reviewed and deemed ethically acceptable by the institutional review board. As most participants were minors aged <18 years, extra ethical precautions were exercised. For participants aged <18 years, written informed consent was obtained from parents or their legal guardians and assent from the adolescents. The assent forms were read in age-specific terms so the minors would clearly understand the purpose of the study, procedures to be followed, risks and benefits, if any, and their rights, such as withdrawal from the study at any time without any penalty. Parents and guardians received full written and verbal explanations of the study, including confidentiality protocols and data privacy protections, so that participation would be based on fully informed decision-making. All data were anonymized and stored securely to protect participant privacy and confidentiality. Identifying information was stored separately from the main dataset and was accessible only to authorized research team members. No financial incentives or rewards were promised to the participants or their families for participating in this study, so as to avoid coercion or undue influence.

## Results

### Age, Usability, and Effectiveness

The study found that age was slightly correlated with app usability and effectiveness. Older adolescents (aged 17‐19 years) generally reported higher usability scores and perceived greater effectiveness than younger participants (Table 1). This could be due to increased digital literacy and maturity in using therapeutic tools among older users, who may navigate the app more intuitively and apply CBT techniques more consistently.

**Table 1.** Demographic characteristics (N=40) and correlation with usability and effectiveness outcomes for the CBT^a^ app.

Characteristic	Participants, n (%)	Usability score, mean (SD)	Effectiveness score, mean (SD)	Preferred features	Notable observations
Age (years)					
13‐15	15 (38)	3.2 (0.5)	3.4 (0.6)	Gamification and mood tracking	Less consistent engagement but reported mood improvements
16‐19	25 (62)	4.1 (0.4)	4.3 (0.5)	Journaling and thought reframing	Higher digital literacy correlated with better usability
Gender					
Male	18 (45)	3.6 (0.7)	3.5 (0.8)	Gamification and relaxation techniques	Gamification boosted engagement; preferred reward-based elements
Female	22 (55)	4.0 (0.6)	4.2 (0.7)	Journaling and mood tracking	Consistent journaling and tracking helped manage emotions
Mental health diagnosis					
Anxiety	20 (50)	4.0 (0.5)	4.1 (0.6)	Mood tracking and relaxation techniques	Frequent mood tracking to manage anxiety triggers
Depression	15 (38)	3.7 (0.6)	3.8 (0.5)	Journaling	Journaling as a primary emotional outlet
Both	5 (12)	3.5 (0.7)	3.6 (0.8)	Combination of all features	Mixed feature use, desire for more personalization
Previous CBT experience					
Yes	10 (25)	4.2 (0.4)	4.3 (0.6)	Thought reframing and relaxation	Higher familiarity led to easier app navigation
No	30 (75)	3.5 (0.6)	3.6 (0.7)	Mood tracking and gamification	Accessibility for beginners; tutorial recommended

a CBT: cognitive behavioral therapy.

### Gender Differences

Gender differences were evident in feature engagement and overall satisfaction with the app. Female participants reported using journaling and mood tracking more frequently and found these features particularly beneficial for managing their emotions. Males, however, demonstrated a stronger preference for gamification elements, suggesting they might respond better to reward-based interactions within the app. This result indicates that gender-sensitive adaptations, like balancing gamified elements with reflective exercises, could optimize engagement.

### Mental Health Diagnosis

Diagnosis type influenced how participants interacted with different app features. Adolescents diagnosed with anxiety frequently engaged with mood tracking to monitor their anxiety triggers and reported that it helped them feel more in control. In contrast, participants with depression found journaling more beneficial, as it provided an outlet for emotional expression. Those with both anxiety and depression reported mixed results, finding both features helpful but expressing a need for more personalized guidance. These findings suggest that tailoring app features based on specific mental health diagnoses may enhance effectiveness.

### Previous CBT Experience

Prior CBT experience was associated with higher usability scores. Participants familiar with CBT concepts found the app easier to navigate and were able to engage more readily with tools like thought reframing and relaxation exercises. Those without prior CBT experience still reported short-term improvements, indicating that the app is accessible for beginners, though they noted that a brief tutorial on CBT basics could improve initial engagement.

### Interaction Between Characteristics

When analyzing the interaction between age and diagnosis, older adolescents with anxiety showed a unique pattern of consistent engagement with mood tracking and relaxation exercises, possibly due to increased awareness of their symptoms and the therapeutic benefits of tracking. This contrasts with younger adolescents with depression, who were less consistent with engagement but reported substantial short-term mood improvements when they did engage, highlighting age and symptom-specific usage patterns.

### Usability Challenges

#### Overview

Several usability challenges were identified through participant feedback (Table 2). While the majority of adolescents found the app’s interface visually appealing, many encountered issues related to its design and navigation. For example, one participant stated, “It was hard to find the tools I needed; sometimes I got lost in the app” [Participant 3, female, 16 years old].

**Table 2.** Themes identified in usability challenges (N=40).

Usability challenge	Description	Values, n (%)
Complex navigation	Difficulty in finding features or completing multistep tasks	20 (50)
Interface design	Issues with text size, button placement, or layout on mobile devices	12 (30)
Content overload	Feeling overwhelmed by the amount of content in some sections	8 (20)

#### Complex Navigation

Of the 40 participants, 20 (50%) reported difficulty navigating through the app, particularly when attempting to access multistep CBT modules. They expressed a desire for clearer instructions and a more simplified user interface.

#### Interface Design

Of the 40 participants, 12 (30%) mentioned that the app’s text size and button placement were not user-friendly, especially when using smaller mobile devices. This affected their overall experience and led to frustration in some cases.

#### Content Overload

Of the 40 participants, 8 (20%) felt overwhelmed by the amount of information presented in certain sections of the app. They indicated that the extensive content occasionally discouraged further use.

### Engagement

Participant engagement with the app varied and was influenced by several factors, including personal relevance and gamification features (Table 3).

**Table 3.** Factors influencing engagement with the CBT^a^ app (N=40).

Factor	Description	Values, n (%)
Motivation (personal relevance)	Continued use linked to personal mental health needs and recognition of the app’s benefits	20 (50)
Gamification and rewards	Positive response to interactive features and rewards	10 (25)
Personalization	Engagement hindered by lack of tailored content and goals	10 (25)

a CBT: cognitive behavioral therapy.

#### Motivation and Personal Relevance

Of the 40 participants, 20 (50%) who found the app’s content personally relevant and aligned with their mental health needs reported higher levels of engagement. These participants were more likely to use the app consistently throughout the 4-week period. For example, one participant noted, “The rewards made it fun and kept me coming back” [Participant 5, male, 17 years old].

#### Gamification and Rewards

Of the 40 participants, 10 (25%) responded positively to the app’s gamified elements, such as rewards for completing exercises and interactive features like mood tracking. They reported that these features enhanced their motivation to continue using the app.

#### Lack of Personalization

Of the 40 participants, 10 (25%) noted that the absence of personalization options, such as customized goals or tailored content, negatively impacted their long-term engagement with the app.

### Perceived Effectiveness

The majority of participants reported experiencing positive mental health outcomes, although the duration of the benefits varied (Table 4). For example, one participant stated, “The app gave me exercises that helped calm me down before an exam” [Participant 2, male, 14 years old].

**Table 4.** Perceived effectiveness of the app (N=40).

Effectiveness outcome	Description	Values, n (%)
Mood improvement	Participants reported a reduction in anxiety or improved mood after using the app	15 (38)
Learning coping skills	Users noted acquiring new coping mechanisms for managing stress and negative thoughts	12 (30)
Short-term benefits	Short-term improvements, but benefits were not sustained without continuous app use	13 (32)

#### Mood Improvement

Of the 40 participants, 15 (38%) reported a noticeable reduction in anxiety levels and an improvement in their mood after completing CBT exercises, particularly those focused on breathing techniques and cognitive restructuring.

#### Learning Coping Skills

Of the 40 participants, 12 (30%) highlighted that they learned new coping mechanisms, such as identifying and challenging negative thought patterns, which helped them manage day-to-day stress.

#### Short-Term Benefits

Despite these positive outcomes, 13 (32%) out of 40 participants mentioned that the improvements were short-term and did not last without regular app usage. This suggests that sustained engagement is necessary for long-term benefits.

### Barriers to App Usage

Several barriers were identified that limited participants’ consistent use of the app or reduced its perceived effectiveness (Table 5).

**Table 5.** Barriers to app usage (N=40).

Barrier	Description	Values, n (%)
App functionality issues	Technical problems like slow loading times or app crashes	8 (20)
Lack of personalization	Limited customization options for individual needs	14 (35)
External distractions	Schoolwork, social media, or lack of time impacting regular usage	18 (45)

#### App Functionality Issues

Of the 40 participants, 8 (20%) experienced technical problems, such as slow load times or occasional crashes, which discouraged them from using the app regularly.

### Lack of Personalization

As previously mentioned, 14 (35%) out of the 40 participants felt that the lack of individualized content limited their overall engagement and the app’s relevance to their specific mental health needs. One participant shared, “I felt like the app understood what I was going through, and that kept me using it” [Participant 7, male, 15 years old].

#### External Distractions

Of the 40 participants, 18 (45%) cited external distractions, such as schoolwork, social media, and general time constraints, as reasons for inconsistent app usage. They suggested that push notifications or reminders could help them stay on track with their CBT exercises. A participant commented, “Knowing others were going through the same thing made me feel less alone” [Participant 4, female, 16 years old].

### Data Analysis Process

A thematic analysis was used to systematically explore the data gathered from focus groups and individual interviews. This qualitative approach is particularly suited to understanding the nuanced experiences of participants, as it allows for the identification of patterns and themes within textual data [40]. The analysis followed an inductive approach, where themes were derived directly from the data without imposing pre-existing frameworks, ensuring that the findings reflect participants’ perspectives authentically [41].

### Steps of Thematic Analysis

The steps of thematic analysis were as follows:

1. Data Familiarization: all interview transcripts were thoroughly reviewed to immerse the research team in the data and gain an initial understanding of the content.
2. Coding: initial codes were generated to highlight recurring patterns, unique responses, and significant statements related to usability, engagement, and perceived effectiveness.
3. Theme Development: codes were then grouped into broader themes that encapsulated the key insights, such as barriers to engagement, facilitators of usability, and outcomes of perceived effectiveness.
4. Review and Refinement: themes were refined iteratively to ensure they were distinct, relevant, and reflective of the dataset as a whole.

This method is appropriate for the study as thematic analysis provides flexibility in analyzing diverse qualitative data and is well suited for understanding user experiences with interventions such as mobile CBT apps [42]. It enables researchers to capture both explicit content and latent meanings in the data.

### Adequacy of Sample Size

The sample size for this study is adequate based on qualitative research standards. Data saturation—a point where no new themes or insights emerge—can often be achieved with 6‐12 interviews, depending on the study’s scope and participant homogeneity [43]. In this study, combining focus groups and individual interviews ensured a rich dataset that captured a range of perspectives while adhering to the principle of saturation.

## Discussion

### Principal Findings and Interpretation

This study explored the usability, engagement, and perceived effectiveness of a mobile CBT app designed for adolescents facing mental health challenges, such as anxiety and depression. The findings provide valuable insights into the potential of mobile CBT apps while highlighting areas for improvement, especially for adolescents in low-resource settings like Iraq.

### Usability and Personalization

One key finding was the usability challenges reported by participants, including complex navigation and nonintuitive design. Participants frequently mentioned difficulties in locating tools or navigating through the app’s interface. This underscores the need for developers to prioritize user-centered design that is simple, clear, and adaptable to different devices and screen sizes.

Features such as guided tutorials and step-by-step walkthroughs can facilitate easier navigation for adolescents with varying levels of digital literacy. Additionally, the app should be rigorously tested across multiple devices to ensure smooth performance and minimize technical barriers that deter consistent use.

A significant recommendation based on the feedback is the incorporation of personalization features, which could enhance the app’s effectiveness and user engagement. Developers could implement adaptive algorithms that adjust content based on user input and progress, allowing the app to deliver more targeted interventions. For example, users could set personal mental health goals, such as reducing anxiety before exams, and the app could tailor CBT exercises accordingly to align with these objectives. Additionally, personalized content delivery based on self-assessment responses could ensure that users receive modules or exercises most relevant to their emotional state or specific stressors. Regular mood check-ins could further refine these recommendations, offering targeted suggestions that align with the user’s evolving needs and experiences.

Consistent with earlier studies, such as Zhang et al [44], the app demonstrated potential in improving adolescents’ mental health outcomes by providing accessible and flexible therapeutic tools. Previous work has highlighted the importance of user-friendly interfaces in promoting app adherence [45], and this study reinforces those conclusions. Participants’ feedback about challenges in navigating the app echoes findings from Cheng et al [46], where adolescents reported that complex interfaces reduced their motivation to engage consistently.

Furthermore, the study aligns with the conclusions of Miller et al [47], which emphasized the critical role of personalization in enhancing the efficacy of digital interventions. Like Milleret al [47], our results suggest that tailoring app content to users’ unique needs and progress is key to maintaining engagement and achieving desired outcomes.

The study revealed that engagement was strongly influenced by participants’ perceptions of the app’s relevance to their specific mental health needs. Participants who felt that the app effectively addressed their challenges were more likely to use it consistently, a finding supported by Banneyer et al [48], who demonstrated that content alignment with user needs significantly improves adherence to digital mental health interventions. Gamification features, such as badges, levels, and progress tracking, were identified as key motivators that sustained interest and encouraged continued use. These findings align with Ng and Wong [49], who reported that gamification elements enhance user engagement by creating a sense of achievement and progress.

Additionally, social features like anonymous peer support or community forums emerged as promising strategies for fostering connection and reducing feelings of isolation. This observation resonates with Silberg et al’s [50] findings, who highlighted the role of social support mechanisms in increasing the acceptability and usage of mental health apps. In collectivist cultures like Iraq, where community and social bonds are deeply valued, enabling adolescents to share their progress or experiences anonymously could enhance engagement by leveraging these cultural strengths. This aligns with Patel et al [51], who noted the importance of culturally sensitive features in ensuring the success of digital health interventions. Future research should investigate the impact of such social features in different cultural contexts to determine their broader applicability and effectiveness.

### Perceived Effectiveness

The perceived effectiveness of the app was influenced by its ability to address specific mental health challenges faced by participants. Such feedback highlights the importance of targeted interventions that address adolescents’ real-world stressors. Participants also emphasized the need for evidence-based tools that provide tangible benefits. Incorporating features that explain the rationale behind each exercise and how it aligns with CBT principles could enhance perceived effectiveness and build user trust.

The findings align with prior research on adolescent mHealth engagement, such as those of Ghosh et al [52] features like personalization, gamification, and caregiver endorsement are critical for adolescent engagement in mHealth apps. Similarly, Oakley-Girvan et al [53] emphasized the importance of adaptive content and user-centered design in sustaining engagement. These studies underscore the importance of tailoring mHealth interventions to meet the unique needs of adolescent users while addressing cultural and contextual factors.

### Long-Term Benefits and Sustainability

While most participants reported short-term improvements in mood and anxiety, the benefits were not sustained without regular app usage. This finding highlights the need for future mobile CBT apps to incorporate features that foster long-term engagement and sustained mental health improvements. Features such as regular reminders, progress tracking, and reinforcement of positive behaviors over time could play a crucial role in maintaining user involvement. For instance, push notifications reminding users to complete CBT exercises, celebrate milestones, or provide motivational messages have been shown to improve engagement and adherence in similar interventions [51].

Additionally, there is significant potential to integrate digital and human support in future app iterations. Adolescents could benefit from a hybrid model that combines the self-guided app with access to mental health professionals or peer mentors when needed. This approach aligns with findings from Gentry et al [54], which demonstrated that blending digital tools with human interaction significantly enhances mental health outcomes. Similarly, Patel et al [51] emphasized that hybrid models are particularly effective in resource-limited settings, where direct access to therapists is scarce. In regions like Iraq, where mental health resources are constrained, a blended approach could address both accessibility and sustainability challenges. By combining the strengths of digital and human support, mobile CBT apps could ensure broader and more enduring benefits, providing an effective solution for sustaining mental health improvements over time.

### Implications for Future Development

The findings of this study highlight several key recommendations for the future development of mobile CBT apps for adolescents, particularly in low-resource settings like Iraq:

- Simplify navigation to enhance usability, ensuring the app is easy to use across different devices and levels of digital literacy.
- Incorporate adaptive personalization that tailors content based on individual user needs, goals, and progress.
- Gamify engagement to sustain motivation and encourage regular use, with a focus on rewards that resonate with the adolescent user base.
- Address external barriers such as distractions and time constraints by providing flexible features like push notifications and offline accessibility.
- Consider cultural and regional factors when designing apps, ensuring that features such as peer support align with the social values of the target audience.

### Recommendations

#### Enhance Personalization

##### Adaptive Content Delivery

Integrate artificial intelligence–based algorithms to tailor content to the user’s progress, mood, and engagement level. For example, adaptive modules can adjust the difficulty of activities based on the user’s performance or offer reminders tailored to individual schedules.

##### User-Centered Goal Setting

Allow users to set personal goals within the app, such as managing daily stress or improving sleep. Personalized progress tracking and feedback can enhance motivation and sustain engagement over time.

##### Localized and Culturally Relevant Content

Incorporate content that resonates with the cultural background and life experiences of adolescents in Iraq, such as language options and culturally sensitive scenarios that foster user connection and comfort.

### Incorporate Gamification Elements

#### Reward Systems and Badges

Introduce badges, points, or achievements for completing exercises, engaging regularly, or reaching personal milestones. A reward system can make engagement feel more rewarding and less clinical.

#### Interactive Challenges and Quests

Design challenges that encourage users to engage with the app routinely, such as completing daily mindfulness exercises or participating in weekly reflections. This can add a playful dimension to the app while reinforcing positive habits.

#### Social Sharing Features

Users can share their achievements with peers or within safe, moderated groups. Social elements can increase accountability, foster community, and provide an additional layer of engagement.

### Streamline Navigation and Interface Design

#### Simplified User Flows

Minimize the steps required to reach core functionalities like exercises or mood tracking. Intuitive navigation should allow users to quickly locate and engage with tools, even if they only have a few minutes available.

#### Onboarding Tutorials

A brief, interactive onboarding process can familiarize new users with app functions, making it easier to navigate from the start. Offering short tutorials on new features introduced in updates can also enhance usability.

### Improve Technical Stability

#### Offline Functionality

Given connectivity challenges, especially in rural areas, consider building offline capabilities for essential features, allowing users to complete exercises or track their mood without internet access.

#### Regular Testing and Updates

Frequent testing for bugs, usability issues, and prompt updates will improve overall stability and user satisfaction.

### Conclusions

Overall, this study demonstrates that mobile CBT apps hold great promise for improving adolescent mental health, particularly in low-resource settings like Iraq. By addressing usability challenges, enhancing engagement through personalization and gamification, and overcoming barriers to usage, mobile CBT interventions can become more effective and accessible tools for young people. As digital health solutions continue to evolve, developers should prioritize user-centered, flexible designs that cater to the unique needs of adolescents in diverse cultural contexts. Future research should explore how to extend the long-term impact of these interventions, potentially through hybrid models that combine self-guided app use with professional support.

## Abbreviations

CBT: cognitive behavioral therapy
mHealth: mobile health
